# Seasonal habitat use by Elephants (*Loxodonta africana*) in the Mole National Park of Ghana

**DOI:** 10.1002/ece3.2962

**Published:** 2017-04-18

**Authors:** George Ashiagbor, Emmanuel Danquah

**Affiliations:** ^1^ Department of Wildlife and Range ManagementKwame Nkrumah University of Science and TechnologyKumasiGhana

**Keywords:** African elephant, elephant distribution, Geographic Information Systems, MaxEnt, Mole National Park, Species Distribution Modeling

## Abstract

To avoid unnecessary waste of limited resources and to help prioritize areas for conservation efforts, this study aimed to provide information on habitat use by elephants between the wet and dry seasons in the Mole National Park (MNP) of Ghana. We compiled coordinates of 516 locations of elephants’ encounters, 256 for dry season and 260 for wet season. Using nine predictor variables, we modeled the probability of elephant's distribution in MNP. We threshold the models to “suitable” and “nonsuitable” regions of habitat use using the equal training sensitivity and specificity values of 0.177 and 0.181 for the dry and wet seasons, respectively. Accuracy assessment of our models revealed a sensitivity score of 0.909 and 0.974, and a specificity of 0.579 and 0.753 for the dry and wet seasons, respectively. A TSS of 0.488 was also recorded for the dry season and 0.727 for the wet season indicating a good model agreement. Our model predicts habitat use to be confined to the southern portion of MNP due to elevation difference and a relatively steep slope that separates the northern regions of the park from the south. Regions of habitat use for the wet season were 856 km^2^ and reduced significantly to 547.68 km^2^ in the dry season. We observed significant overlap (327.24 km^2^) in habitat use regions between the wet and dry seasons (Schoener's D = 0.922 and Hellinger's‐based I = 0.991). DEM, proximity to waterholes, and saltlicks were identified as the key variables that contributed to the prediction. We recommend construction of temporal camps in regions of habitat use that are far from the headquarters area for effective management of elephants. Also, an increase in water point's density around the headquarters areas and selected dry areas of the park will further decrease elephant's range and hence a relatively less resource use in monitoring and patrols.

## 1 Introduction

**Figure 1:**
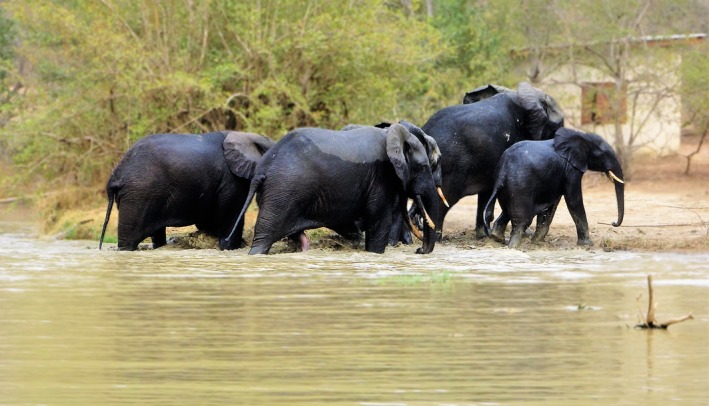
Picture of African elephant (*Loxodonta Africana*) in the headquarters area of Mole National Park (Picture credit: Gilchrist S. Darko, Dept. of Wildlife and range Management, KNUST)

The African elephant (*Loxodonta Africana*) Figure 1 is the largest living terrestrial mammal and occurs widely across Africa. Nevertheless, their population and total elephant range in Africa has decline over the years. In West Africa, their numbers have declined considerably resulting in listing as Vulnerable, on the International Union for Conservation of Nature (IUCN) Red List of Threatened Species (Thouless et al., 2016). In North Africa, they have been extinct since the European middle ages (UNEP et al., 2013).

The smallest and fragmented populations of *L. africana* can be found in sub‐Saharan Africa, mostly in tropical swamp forest, Savannah, and desert habitats and they tend to extend their habitats in search of forage, water, and cover (Blanc et al., 2007; UNEP, 2013; Mwambola et al., 2016). The 2016 IUCN African Elephant Status Report listed nine areas in Ghana with elephant populations. Five of these areas have extremely small populations less than 100 elephants in the forest and Savannah habitats. The remaining two occurs in the forest‐Savannah transition zone (Thouless et al., 2016; Danquah & Oppong, 2014). Mole National Park (MNP) holds the largest population of elephants in the Savannah habitat zone of Ghana (Ghana Wildlife Division, 2000; Thouless et al., 2016).

The choice of habitat use by an animal within a complex and dynamic landscape is a central theme in conservation ecology (Hull et al., 2016). There are a number of proximate factors that guides an elephant's decision to reject or select a particular habitat type. Forage availability, water, land cover, and topographic characteristics of a landscape are considered fundamental factors inducing habitat selection by elephants (Ananda Kumar, Mudappa, & Shankar Raman, 2014; Sukumar, 2003). However, the ranging behavior of elephants is obviously influenced by surface water and forage availability of a certain type and quality (Sukumar, 2003). Sukumar (2003) outlined how specific diets, nutrition, and foraging strategies of different elephants groups may affect their choice of habitat use.

Whiles these variables remain evident in the literature, park specific habitat mapping is invaluable in conservation and management decision of a protected area. Nevertheless, fine‐scale data on the distribution and habitat utilization of elephants and the environmental variables that affect their distribution is a major constraint in the MNP. No information exists on the habitat use of elephants in MNP. For conservation and planning purposes, basic information on elephants’ spatial distribution across seasons is very important (Hedges, 2012). Knowledge of niche of elephants in MNP can help avoid unnecessary waste of conservation resources and help prioritize specific areas for conservation efforts (Lin et al., 2008). The identification of these priority regions in MNP and the key environmental variables that defines these regions are essential if effective conservation action and habitat management are to be implemented (Babaasa, 2000; Platts et al., 2010).

The availability of Geographic Information Systems (GIS) software can help explore the seasonal distribution and habitat use by elephants in MNP. Elith et al. (2006) identified Maximum Entropy Modelling tool (MaxEnt) as a better performing Species Distribution Modeling (SDM) tool than the more widely used DOMAIN and Genetic Algorithm for Rule‐set Prediction (GARP). MaxEnt is a species distribution model (SDM) intended for presence‐only distribution modeling, and its predictive performance is competitive with other methods (e.g., generalized linear or additive models) (Elith et al., 2011; Phillips, Anderson, & Schapire, 2006; Phillips, Dudík, & Schapire, 2004). MaxEnt and GIS tools have been used in recent times in SDM and in understanding the ecological niche of species (Barnhart & Gillam, 2014; Buffum, McGreevy, Gottfried, Sullivan, & Husband, 2015; Fourcade, Engler, Rodder, & Secondi, 2014; Hof, Jansson, & Nilsson, 2012; Junker et al., 2012; Martínez‐Freiría, Tarroso, Rebelo, & Brito, 2016; Papeş & Gaubert, 2007; Phillips et al., 2004, 2006; Puschendorf et al., 2009; Williams et al., 2009).

In this study, we aim to examine the habitat use by elephants in the MNP using GIS and MaxEnt between the wet and dry seasons. Given that water availability in the MNP varies between the wet and dry seasons, we hypothesized that elephant's habitat use in MNP will vary between the wet and the dry seasons.

## 2 Methods

### 2.1 Study area

**Figure 2:**
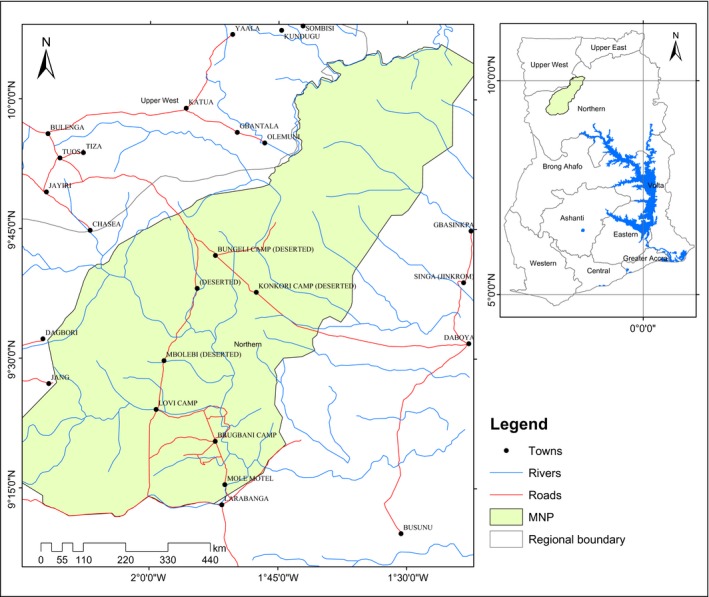
Map of Mole National Park (MNP)

MNP is the largest protected area in Ghana situated in the heart of the pristine Guinea Savannah Woodland vegetation in northern Ghana and covers approximately 4,850 km^2^. It lies between latitudes 9°12′N and 10°12′N and longitudes 1°20′W and 2°15′W (Figure 2). The park forms part of the White Volta catchment, with networks of rivers and streams which drains into the White Volta with Mole and Lovi being the major ones. The vegetation type is characterized by widespread tall grasses which are interspersed with deciduous acacia trees and other trees. Also occurring along the active zones of the major rivers and streams is the closed Savannah riparian vegetation cover (Mole National Park, 2015). The most dominate land cover in the park is the open woodland Savannah with grass under growth and open grassland found on areas with shallow soils (Burton et al., 2011; Dankwa‐Wiredu & Euler, 2002).

The Park has the most viable unique breed of elephant population in Ghana, which are not hostile, and aggressive, compared to other elephant populations in the rest of Africa. As such, MNP is considered a hot spot for tourism and elephant conservation (Mole National Park, 2015). An aerial survey in 2007 by Bouché (2007) estimated 401 elephants in the park with a density of 0.08 elephants/km^2^ and mean group size of 9.11 ± 14.66 (*SD*); herds ranged up to 80 individuals. The average temperature in the Park is about 28°C, falls to 26°C in December, and rises to 31°C in March. Two seasons are observed in the park. The dry season starts in November and ends in March and the wet from April to October (Mole National Park, 2015).

### 2.2 Elephant data collection

We compiled the coordinates of all elephant encountered between 2012 and 2014. Data of encounter rates through field patrols were obtained from management of MNP. As a protected area, conventional law enforcement was used in the form of foot patrols, starting from the range camps, including the park headquarters. Recordings of the number of patrol staff, exact patrol duration, area patrolled, and the number of different large mammal species encountered and their locations as a form of standardization were kept by the management of the park. In this study, we extracted only data for elephants, specifically their GPS locations and season (months) of patrol. In all we extracted a total of 516 GPS coordinates, 256 for dry season (November to March), and 260 for wet season (April to October). The data were clipped to the MNP boundary despite some elephant sightings outside the park. This is because our analysis focused solely on the space utilization and selection within the park.

### 2.3 GIS mapping and data preparation

Our overall approach was to generate land cover, Normalised Difference Vegetation Index (NDVI), digital elevation model (DEM), slope, aspect, terrain ruggedness and proximity to water sources, roads, and human settlements/camp sites as predictor variables for the model. We selected these variables based on the ecological knowledge of the species and prior work (Chamaille‐Jammes, Valeix, & Fritz, 2007; De Boer, Ntumi, Correia, & Mafuca, 2000; De Knegt et al., 2011; Graham, Douglas‐Hamilton, Adams, & Lee, 2009; Harris, Russell, van Aarde, & Pimm, 2008; Kyale, Ngene, & Maingi, 2011; Ochieng, 2015). Meteorological variables (i.e., precipitation and temperature) were not considered in this model because the study catchment spans within the same climatic zone with constant climatic distribution. Also there is only one meteorological station in the catchment region used to generalize temperature and precipitation in the MNP.

We used NDVI as a measure of spatiotemporal patterns in vegetation productivity and forage availability in the entire park (Bohrer, Beck, Ngene, Skidmore, & Douglas‐Hamilton, 2014; Young, Ferreira, & van Aarde, 2009). To extract NDVI for the study area, we obtained readily available Landsat 8 Operational Land Imager (OLI) datasets from USGS Earth Explorer for both wet and dry seasons. Two images were obtained for the dry season and three images for the wet season. The choice of images was based on availability and absence of cloud cover between 2012 and 2014. To calculate NDVI, we first converted DN values to top of atmosphere (TOA) reflectance using the following equations adopted from U S Geological Survey (2016).

$$\rho_{\lambda }=\frac{M_{\rho }Q_{\mathrm{cal}}+A_{\rho }}{\text{sin}(\theta )}$$

where

$\rho_{\mathrm{OLI}\;\mathrm{Band}\;i}$ = TOA Reflectance for OLI Band i

*M*_ρ_ = Reflectance multiplicative scaling factor for the band = 0.00005

*A*_ρ_ = Reflectance additive scaling factor for the band = −0.1

*Q*_cal_ = Quantized and calibrated standard product pixel values (DN)

θ = Local sun elevation angle = 54.33.

The values for reflectance multiplicative and additive scaling factors and the local sun elevation angle were obtained from the metadata file that came along with each OLI data.

OLI data Band 4 (VIS:RED) and Band 5 (NIR) were used as Inputs for Q_cal_

We calculated the NDVI values for the study area from the calculated TOA reflectance for Band 4 and Band 5 using the following formula.

$$\text{NDVI}=\frac{\rho_{\mathrm{OLI}\;\mathrm{Band}\;5}-\rho_{\mathrm{OLI}\;\mathrm{Band}\;4}}{\rho_{\mathrm{OLI}\;\mathrm{Band}\;5}+\rho_{\mathrm{OLI}\;\mathrm{Band}\;4}}$$

The NDVI values were calculated separately for both dry and wet seasons’ satellite images. The NDVI images calculated were then averaged separately for the wet season and the dry season months using the Cell Statistics tool in ArcGIS (Sibanda et al., 2016).

To obtain a land cover for the park, we classified the wet season Landsat 8 Operational Land Imager (OLI) datasets. We collected 145 training samples through field GPS visits and Google earth images to aid with the supervised classification. Using supervised classification system, specifically using the maximum‐likelihood algorithm, we classified the satellite image into four land cover classes (Ashiagbor & Laari, 2013; Kabba, Li, Tamba, & Kabba, 2011; Toosi, Fakheran, & Soffianian, 2012). We examined the values of NDVI to identify pixels that corresponds to no vegetation (very low and negative values). Using these identified pixels, we replaced the erroneously classified classes to improve the accuracy of our classification using the knowledge‐based classifier tool, an approach adopted from Vîjdea, Sommer, and Mehl (2004) and Schimmer (2008). Kappa coefficient, overall accuracy, user's accuracy, and producer's accuracy were selected as measures to assess the accuracy of the classification. This was performed using 85 validation GPS points over the study area. We obtained an overall accuracy of 81.2% for the catchment (see Appendix 1 for results of accuracy assessment).

The dominant land cover types mapped (see Appendix 2 for land cover map) in the park are sparse woodlands/shrubs (open woodland) with under growth Savannah grasses constituting 2,057.58 km^2^ (42.47%). The open woodland gives way to the more open areas of the Savannah grassland, which covers an area of 1,816.95 km^2^, representing 37.51% of the park's land cover. In the wet season, the grassland can grow as long as 3 m and withers in the long dry season. The closed canopy woodland, with a total area of 873.86 km^2^, is mainly seen along the river networks and also on the high elevation areas toward the northern section of the park. Built‐up/bare areas formed the least land cover type representing just about 1.98% of the park. Built‐up and bare areas are mainly the headquarters area and some camp sites within the park. Open rock surfaces also constitute this cover type.

For elevation, we downloaded the global 1‐arcsecond (30‐m) SRTM digital elevation model (DEM) for the study catchment from the United States Geological Survey's Earth Explorer site (http://earthexplorer.usgs.gov/). Using the Raster conversion tool in ArcGIS, we calculated slope gradient and aspect to obtain our slope and aspect raster surfaces. We computed the terrain ruggedness index (TIR) in ArcGIS using the formulae proposed by Riley, DeGloria, and Elliot (1999). The DEM downloaded for MNP reveals elevation ranging from 122 m above mean sea level and increases gradually from the edges of the study area to the central areas of the park to an elevation of 486 m. The highest elevation areas cut diagonally from the northern parts of the park to the central opposite end of the park separating the park into two sections. The slope analysis reveals a generally gentle slope with over 90% of the park with gradient below 4% (see Figure 4 for DEM and Slope maps).

We used proximity to streams and water holes in MNP to understand how water availability in the park influences habitat selection and use by elephants. Rainfall is the major determinant of water availability in MNP. Most of the streams in the park are seasonal and dries out completely in the dry season, leaving pockets of waterholes. For this reason, we mapped out separately streams that were perennial and streams that were only active in the wet season (April to October). We created separate Euclidian distance maps for dry season proximity to streams and wet season proximity to streams (Muposhi et al., 2016). The stream network vector used was digitized from existing topographic map of the park and validated through field visits and Google earth images. GPS locations of water holes were obtained from park managers and updated through field survey and Google image sources. We recorded a total of seventy‐seven waterholes (four artificial holes inclusive) in the wet season. These holes occurred along the major stream networks in the park. In the dry season, most of the waterholes dry up leaving only twenty‐one (21) waterholes. Sometime even in severe dry seasons (March), most of these waterholes completely dry up leaving just but a few and the artificial waterholes. With these waterholes data, we calculated the Euclidean distance for the water holes in the catchment to produce proximity to water holes’ raster separately for the wet and dry seasons (Muposhi et al., 2016).

To describe anthropogenic influences on elephant's distribution in the park, we used proximity to camp sites and roads calculated from the Euclidean distance spatial analysis tool in GIS (Rood, Ganie, & Nijman, 2010). Roads networks and camp sites within the park were digitized from Google earth images.

### 2.4 Data preparation

Preliminary analyses were performed to test for collinearity of predictor variables. We used the band collection statistics tool in ArcGIS to provide the multivariate analysis of all predictor raster variables extracted to obtain the correlation matrices (Appendix 3). We exempted the predictor variables that had a strong correlation (r > .8) (Fourcade et al., 2014; Jarnevich & Reynolds, 2010). TRI and proximity to roads were therefore exempted from the analysis because they correlated strongly with slope and proximity to saltlicks, respectively.

### 2.5 Elephants habitat use mapping using

We modeled the seasonal habitat use of *L. africana* using the MaxEnt version 3.3.3k. We adopted the methodology for the elephants’ distribution modeling from the following publications (Barnhart & Gillam, 2014; Buffum et al., 2015; Fourcade et al., 2014). We selected pseudo‐absence file from inside‐protected area to provide the same bias as the presence location. We divided presence data into 70% for training and 30% for testing and ran the jack‐knife validation function to minimize biases associated with small sample size. For the dry season, 182 presence records were used for training and 77 for testing. For the wet season, 180 presence records were used for training and 77 for testing were used.

We then threshold the final output logistic models to a binary prediction of “suitable” or “nonsuitable” regions of habitat use, using the equal training sensitivity and specificity threshold values (calculated by MaxEnt) of 0.177 and 0.181 for the dry and wet seasons, respectively (Bartel & Sexton, 2009; Maria, 2014; Muposhi et al., 2016). Our choice of equal training sensitivity and specificity threshold values was to balance the accuracy of areas correctly modeled as present and absent in the training and test data (Muposhi et al., 2016). To evaluate the accuracy of the final binary maps, we calculated the sensitivity (Se), specificity (Sp), true skill statistic (TSS) and Kappa statistics (k), well‐accepted accuracy measures (Allouche, Tsoar, & Kadmon, 2006; Liu, White, & Newell, 2011, 2013). However, we used TSS (a special case of kappa) as the measure of accuracy in this study taken into account that TSS is not affected by prevalence, the size of the validation set, and it combines sensitivity and specificity so that both omission and commission errors are accounted for (Allouche et al., 2006). Kappa and TSS values less than 0.4 indicate poor agreement, values between 0.4 and 0.75 indicate good agreement, and values approximately 0.75 and above indicate very good to excellent agreement with 1.0 as a perfect agreement (Monserud & Leemans, 1992).

We performed an intersection analysis to identify similar habitat use (areas of overlap) between the wet and dry seasons. We also marked out habitats that were predicted to be suitable only for the wet and dry seasons, respectively. To test whether the habitat use by elephants in the wet season and the dry season have significant ecological differences, we calculated Schoener's D and Hellinger's‐based I using ENMTools (Warren, Glor, & Turelli, 2008; Zhang et al., 2014). To understand how the seasonal availability of water in MNP affected habitat use and selection, we conducted a Getis‐Ord Gi* spatial hot spot analysis (Sibanda et al., 2016).

## 3 Results

Results from the MaxEnt elephant's distribution modeling reveal an overall good model predictability with a receiver operating characteristic (ROC)‐AUC test scores of 0.883 ± 0.018 and 0.924 ± 0.016, respectively, for the dry season and the wet season (Table 1). Sensitivity of prediction of elephants at a site was 0.909 for the dry season and 0.974 for the wet season and Specificity was 0.579 and 0.753 for the dry and wet seasons, respectively. A TSS of 0.488 was recorded for the dry season and a TSS of 0.727 for the wet season indicating a good model agreement.

**Table 1:** Results showing accuracy assessment of habitat use model and areas of use by elephants for both dry and wet seasons

Season	Area (km^2^)	AUC	Accuracy	Sensitivity	Specificity	k	TSS
Dry	547.68 (11.29%)	0.883	0.582	0.909	0.579	0.308	0.488
Wet	856.00 (17.64%)	0.924	0.754	0.974	0.753	0.413	0.727
Intersection	327.24 (6.7%)						

Table 2 shows results of relative contribution of the nine predictor variables to elephant's distribution in MNP as calculated by MaxEnt. From the model, DEM and proximity to saltlicks and waterholes were identified as the most important contributing predictors of elephant's distribution in the MNP for both the dry and wet seasons. In the wet season, DEM contributed to 30.7%, proximity to saltlicks 28.6%, proximity to waterholes 18.7%, and proximity to camp sites 18.3%. All the other remaining variables contributed to a total below 5% to the habitat use in the wet season. Also in the dry season, DEM and proximity to saltlicks and proximity to water holes were identified as the main predictors of elephant's habitat selection, contributing 43.7%, 29.1% and 14.7%, respectively.

**Table 2:** Relative contributions of the environmental variables to elephants distribution in MNP for dry and wet season model

Variable	Dry season (%)	Wet season(%)
DEM	43.7	30.7
Distance from saltlick	29.1	28.6
Distance from water holes	14.7	18.7
Distance from camp sites	5.5	18.6
Aspect	0.4	1.4
Distance from streams	1.5	0.5
Land cover	1.2	0.8
NDVI	2	0.1
Slope	1.8	1.6

**Figure 3:**
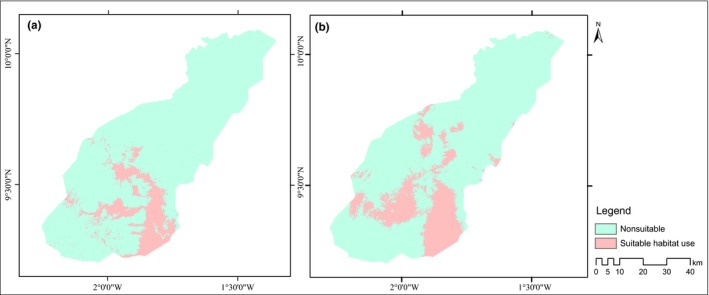
Final output logistic models threshold to binary predictions of suitable habitat use regions using equal training sensitivity and specificity threshold values: (a) dry season and (b) wet season

Figure 3 shows results of final logistic model thresholded to binary predictions of suitable habitat use regions for dry and wet seasons using the equal training sensitivity and specificity threshold values. Visualizing the final model showed that wet season habitat use is larger than the dry season habitat use. The region of habitat use by elephant in the wet season is 856.00 km^2^ representing 17.64% of the total area of MNP. In the dry season, the area of habitat use reduced to 547.68 km^2^ (11.29%). The potential distribution of the elephants in the wet season is 308.32 km^2^ more than the dry season. The regions of habitat use common to both the dry and wet seasons are 327.24 km^2^ (6.7%) (Table 1).

## 4 Discussions

We have investigated how seasonal variations in surface water availability constraints elephant's movement and their choice of habitat use and selection in MNP. Our results reveal 308.32 km^2^ difference in habitat use between the wet and dry seasons. In comparison with the maximum core home range of 31.5 km^2^ of African elephants observed in the Pongola Game Reserve, South Africa by Shannon, Page, Slotow, and Duffy (2006), our observed difference in range is ecologically significant. In the wet seasons, elephants in MNP range over large areas (856.00 km^2^) and significantly reduce their range to 547.68 km^2^ in the dry seasons. The observed significant overlap (Schoener's D = 0.922 and Hellinger's‐based I = 0.991) between the wet and dry seasons was 327.24 km^2^ representing 6.7% of the entire park.

**Figure 4:**
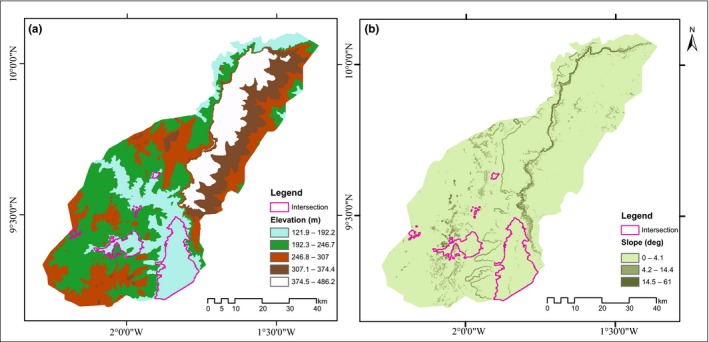
Suitable habitats used by elephants predominantly within lower elevation regions (121.9–192.2 m) of the park and within relatively flat terrains (Slope <4°) for both the dry and wet seasons: (a) elevation map and (b) slope map

These regions of suitable habitat use were observed to be constrained to the central and southern regions of the protected area for both dry and wet seasons (Figure**** 3). This observation agrees with a survey carried out in the park in 2007 by Bouché (2007) and information gathered from park managers which indicate elephants’ presence only within these regions of the park. We identified elevation difference in the park to have accounted for this constraint in range. In MNP, there is a high elevation hill and a relatively steep slope (Slope >14°) that separates the northern section of the protected area from the southern zone. From Figure**** 4 **,** it is evident that the suitable habitats regions fall predominantly within the lower elevation regions of the park with elevation between 121.9 and 192.2 m and within flat terrains (Slope <4°) for both the wet and dry seasons. Consistent with similar studies by Wall, Douglas‐Hamilton, and Vollrath (2006), Lin et al. (2008) and Ochieng (2015), elephants’ density and suitable habitat use regions were found to be constrained by increasing elevation and slope. Wall et al. (2006) explained that, elephants avoid high elevation areas and steep slopes due to the risk of injury. Also, in order to optimize their energy needs, Ntumi, Aarde, Van Fairall, and De Boer (2005) posited that, elephants avoid regions of higher elevation and steep slopes.

**Figure 5:**
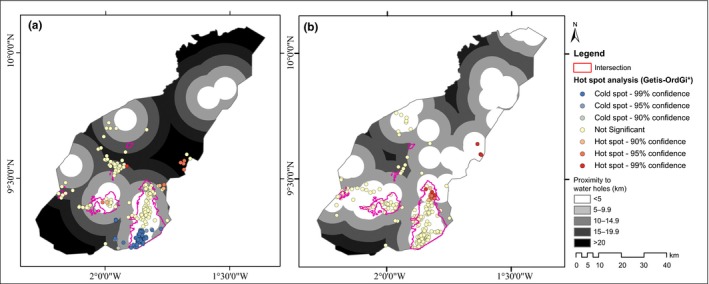
Proximity to water holes’ map showing suitable habitat regions completely within the 5‐km buffer zones of water holes in the park for both the dry and the wet seasons and hotspot clusters: (a) dry season and (b) wet season

The choice of habitat selection and use was a variable of the seasonal variations in surface water availability in the park between the wet and dry seasons. The results of our hotspot clustering analysis reveal no statistically significant clustering (Observed General G = 0.4437, z‐score = 0.097 and *p*‐value = .922) around water holes in the wet season (Figure**** 5b). The abundance of water in the wet season releases the elephants from restrictive movements allowing them more flexibility which may have led to the extension in their range (Cushman, Chase, & Griffin, 2005). In the dry season, however, our hotspot clustering analysis (Observed General G = 0.382, z‐score = −1.753 and *p*‐value = .079) did reveal a statistically significant clustering resulting in cold spots of clustering events. The cold spots clustering is observed within the 5‐km buffer of water holes in the park (Figure**** 5a), indicating that elephants in the dry season limit their range so as to stay closer to the permanent water holes. This explains the reduction in range of elephants in the dry season, keeping in mind that elephants’ ranges are influenced by the distance they need to forage from available water sources (Ross, 2016). Sukumar (2003) confirmed the shrinking of home range size during the dry season and expansion during the wet season. He explained that, when water is scarce, elephants would naturally be confined to small areas where resources are available.

Moreover, most saltlicks (regions of soil where minerals are concentrated) were located in lowland areas close to rivers (Blake et al., 2011) and hence coincided with suitable elephant habitat in MNP. This observation in addition to elephants’ natural affinity for saltlicks could be a contributory factor for the importance of saltlicks (29.1% for dry season and 28.6% for wet season) in predicting habitat use by elephants in MNP. Weir (1972) showed that dependence on woody plants and natural water supplies alone may not be adequate to meet the minimum mineral requirements of elephants. Hence, like many large herbivores, elephants supplement diets from mineral licks. It is worth noting that some water holes and points of high mineral concentrations also occur in the northern regions of the park; however, these regions were still classified nonsuitable habitats. As noted earlier, this may be due to the high elevations and the steep slope that separate the two regions stopping elephants from traversing to those regions.

From Figure**** 5a,b, it is evident that the suitable habitat regions mapped fell completely within the 5‐km buffer zones of water holes in the park for both the dry and the wet seasons. This confirms the importance of water and water availability in the distribution of elephants as demonstrated in publications relating to the niche of elephants (Blake, 2002; Chamaille‐Jammes et al., 2007; De Knegt et al., 2011; Harris et al., 2008; Leggett, 2006; Mwambola et al., 2016; Nellemann, Moe, & Rutina, 2002; Ngene, Skidmore, Van Gils, Douglas‐Hamilton, & Omondi, 2009; Ngene et al., 2010; Ochieng, 2015; Rood et al., 2010; Shannon, Matthews, Page, Parker, & Smith, 2009; Verlinden & Gavor, 1998). The wet season in MNP is characterized by a large expanse of open Savannah grasses, ensuring there is plenty of vegetation (forage). This provides elephants the liberty to range freely across the whole southern regions of the park, hence the increase in regions of habitat use. In the long dry season, however, the ephemeral streams flowing through the park dry up leaving behind a few waterholes. During this period, the grass landscapes turn brown and withers, leaving only areas close to waterholes to sustain forage availability in the park. Hence, elephants tend to congregate more around available forage near to the few waterholes with water in the dry season. These localized forage availability relates to the statistically significant cold‐spots clustering events around waterholes in the dry season. In summary, the results indicate that decreased surface water availability in the dry season, leading to decreased feeding and possible socializing opportunities, could be driving the contraction of elephant dry season range across the MNP taking into account elevation as a potential limiting factor.

An unanticipated result of our analysis was the relatively weak (dry season = 1.5% and wet season = 0.5%) contribution of streams in the park to elephant distribution in the park (Table 2). This could be attributed to the high poaching activity near streams in MNP (not tested in this study) as reported by managers of the park. Compared to the open areas around waterholes, poachers prefer hiding in the closed riverine vegetation surrounding streams where they will not be easily spotted by patrol teams. Sukumar (2003) explained that elephants in their lifetime establishes familiar home ranges, and once this regions are established, movements within them are calculated based on previous experience. Thus, it seems that elephants may be avoiding riverine vegetation due to the apparent insecurity (e.g., poaching) associated with such areas in MNP. It is likely other factors may be contributing to this observation. Hence, determining these factors will be critical in understanding the habitat selection by elephants in the park and also in the management of the MNP.

Also, anthropogenic related activities (distance to roads and camp sites) in the park had little or no consequent effect on elephant distribution in MNP. Contrary to studies by Lin et al. (2008) and Rood et al. (2010) where elephants avoided areas with high human activities, elephants in MNP were most of the time spotted around park's settlement areas with human activities. This is because anthropogenic activities in the MNP are mostly tourist related and are highly controlled by park management therefore have little or no wildlife impact and habitat fragmentation. Other human‐related activities such as farming and cattle grazing were absent in the park as noted in a study by Bouché (2007) and confirmed by park managers. Also, according to Lin et al. (2008) elephants tolerate some levels of human disturbance and activities and therefore are not upset by the activities in the park's settlement areas. We also observed that all of the artificial water holes are found around the park's settlement areas. These water holes attract elephants especially in the dry season when there is scarcity of water in the park. This may have also accounted for the classifications of camp site as suitable regions for elephant's habitation.

## 5 Conclusions

Our research draws attention to matters relating to wildlife monitoring and resource allocation. Park managers in Mole National Park (MNP) conduct regular patrols, surveillance, and monitoring operations against any illegal activities within the park as to safeguard its ecological integrity. A plethora of the literature has demonstrated that curbing poaching and other illegal activity in protected areas predominantly depends on resource allocation for law enforcement, in terms patrol effort and capital. However, funds allocations for protected area in Ghana have been consistently low, limiting the enforcement of wildlife laws and the efficiency of anti‐poaching activities (Jachmann et al., 2011; Myers, Mittermeier, Mittermeier, da Fonseca, & Kent, 2000). Considering the financial constraints, it is therefore important that enforcement operations be carried out cost‐effectively in order to safeguard the already limited financial resources (Jachmann, 2008). Myers et al. (2000) recommended an approach that places premium on prioritization, that is, identify regions featuring exceptional concentrations of endemic species and concentrate resources there. In line with this recommendation, our generated maps of seasonal habitat use by elephant's in MNP will ensure that patrol operations are carried out purposively and cost‐effectively. We recommend citing of temporal camps in these regions of habitat use that are far from the headquarters area for effective management of elephants.

Also, creation of more dams in suitable areas of the park such as what has been recently performed near the Zaina Lodge camp site may be an important tool to manage elephants in MNP. Grainger et al. (2005) submitted that elephants’ home ranges decrease with an increase in water point density. A decrease in home range will imply relatively less resource use in monitoring. This may also induce high elephants citing by tourist and hence an increase in internal revenue generation by the park management. The water holes must, however, be created and spread within the dryer areas of park at short distances. For this, proper scientific techniques should be implemented so that elephants can easily use the resource provided. Nevertheless, conservation managers should consider potential elephant‐induced biodiversity changes, positive or negative, that may arise near the dams or water holes. Managers should also consider other species that may react to dam openings before a provision policy is drafted because it is possible that nontarget species may react to large‐scale water point openings.

## Conflict of interest

None declared.

## Appendix 1

### Accuracy totals

**Table:**

Class name	Reference totals	Classified totals	Number correct	Producers accuracy	Users accuracy
Built‐up/bare areas	5	3	3	60.00%	100.00%
Savannah grassland	37	36	32	86.49%	88.89%
Open Savannah woodlands/shrubs	30	31	24	80.00%	77.42%
Closed canopy woodland	13	15	10	76.92%	66.67%
Total	85	85	69		

Overall Classification Accuracy = 81.18%.

### Kappa (K^) Statistics

Overall Kappa Statistics = 0.7139.

Conditional Kappa for each Category

**Table:**

Class name	Kappa
Built‐up/Bare areas	1
Savannah grassland	0.803
Open savannah woodlands/shrubs	0.651
Closed canopy woodland	0.607

## Appendix 2

### 2.1

FIGURE A2 (a) Land use land cover map of MNP, (b) vegetation productivity and forage availability in MNP for wet season and (c) vegetation productivity and forage availability in MNP for dry season

## Appendix 3

### Collinearity of predictor variables calculated using the Band Collection Statistics tool in ArcGIS

**Table:**

Layer	var1	var2	var3	var4	var5	var6	var7	var8	var9	var10	var11	var12	var13
var2	0.012												
var3	0.08	0.341											
var4	0.268	0.522	*0.862										
var5	0.301	0.027	0.023	0.081									
var6	0.324	0.002	0.017	0.073	0.491								
var7	0.261	−0.045	0.045	0.059	0.158	0.035							
var8	0.342	0.030	0.033	0.089	*0.899	0.338	0.129						
var9	0.194	0.049	0.035	0.063	0.206	0.102	−0.027	0.301					
var10	−0.002	−0.060	−0.082	−0.078	0.024	0.202	−0.128	0.027	0.100				
var11	0.255	−0.042	0.043	0.067	0.059	0.088	0.366	0.074	−0.042	−0.151			
var12	0.037	0.053	0.034	0.016	0.025	0.204	−0.229	0.207	0.484	0.206	−0.113		
var13	0.196	0.092	0.078	0.109	0.023	0.130	−0.106	0.082	0.114	0.15	−0.025	0.300	
var14	0.137	−0.058	0.035	0.027	0.066	0.022	0.768	0.036	−0.074	−0.068	0.437	−0.224	−0.093

var1, DEM; var2, Aspect; var3, Slope; var4, Terrain ruggedness index (TRI); var5, Proximity to Road; var6, Proximity to camp sites; var7, LULC; var8, Proximity to salt lick; var9, Proximity to water hole in dry season; var10, Proximity to stream in dry season; var11, NDVI in dry season; var12, Proximity to water hole in wet season; var13, Proximity to stream in wet season; var14, NDVI in wet season. *variables showing strong correlation.
